# Is a Hospital Pageant Practicable?

**Published:** 1921-05-21

**Authors:** Gilbert G. Panter

**Affiliations:** Secretary. Great Northern Central Hospital, Holloway Road, N.


### IS A HOSPITAL PAGEANT PRACTICABLE?

*To the Editor of* The Hospital.

[The following letter has been sent to the Secretaries of the London hospitals]:

Sir,—The Committee of Management of this hospital, at its meeting in March, recommended the formation of a Voluntary Hospitals Publicity and Propaganda Committee, as announced in the press.

The Hospital, with its usual readiness to further all proposals for the advancement of institutional interests, warmly supported the suggestion, and in its issue of May 7 advocated (as a first step towards making the work of hospitals more widely known among the general public) the holding of a Pageant in which all London voluntary hospitals should combine. This suggestion has appealed to many hospital workers, and, as a means of securing the opinion of hospital secretaries of the Metropolis, I have been asked to convene a meeting to discuss the practicability of the proposal.

I hope that I shall not be thought presumptuous in letting you know that, as a matter of convenience, The Hospital will set aside a room for a meeting at 28 Southampton Street, Strand, at 3.30 p.m. on Friday, the 20th inst., and it is hoped you may be able to be present to give the meeting the advantage of your advice.—Yours faithfully,

Gilbert G. Panter, Secretary.

Great Northern Central Hospital, Holloway Road, N.

May 13, 1921.

